# Rigorous pH measurement in non-aqueous solution: measurement method and reference values in ethanol

**DOI:** 10.1007/s00216-023-05043-5

**Published:** 2023-11-25

**Authors:** Frank Bastkowski, Agnes Heering, Emrah Uysal, Lokman Liv, Ivo Leito, Raquel Quendera, Luís Ribeiro, Lisa Deleebeeck, Alan Snedden, Dániel Nagy, Zsófia Nagyné Szilágyi, Filomena Camões, Bárbara Anes, Matilda Roziková, Daniela Stoica

**Affiliations:** 1 Physikalisch-Technische Bundesanstalt, Bundesallee 100, 38116 Brunswick, Germany; 2 University of Tartu, 14a Ravila Street, 50411 Tartu, Estonia; 3 Electrochemistry Laboratory, Chemistry Group, The Scientific and Technological Research Council of Turkey, National Metrology Institute, (TUBITAK UME), 41470 Gebze, Kocaeli, Turkey; 4 Instituto Português da Qualidade, R. António Gião, 2829-513 Caparica, Portugal; 5 DFM A/S, Kogle Allé 5, 2970 Hørsholm, Denmark; 6 Government Office of the Capital City Budapest (BFKH), Németvölgyi Út 37-39, 1124 Budapest, Hungary; 7 FCiências.ID, Centro de Química Estrutural, Faculdade de Ciências da Universidade de Lisboa, Campo Grande, 1749-016 Lisbon, Portugal; 8 Czech Metrology Institute, Okružní 31, 63801 Brno, Czech Republic; 9 Laboratoire National de Métrologie Et d’Essais (LNE), 1 Rue Gaston Boissier, 75015 Paris, France

**Keywords:** Unified pH scale, Reference values, Non-aqueous buffer, Interlaboratory comparison, Differential potentiometry, Commercial glass electrodes

## Abstract

The recently introduced unified pH (${\mathrm{pH}}_{\mathrm{abs}}^{{\mathrm{H}}_{2}\mathrm{O}}$) concept enables rigorous pH measurements in non-aqueous and mixed media while at the same time maintaining comparability to the conventional aqueous pH scale. However, its practical application is hindered by a shortage of reference ${\mathrm{pH}}_{\mathrm{abs}}^{{\mathrm{H}}_{2}\mathrm{O}}$ values. In order to improve this situation, the European Metrology Research Project (EMPIR) UnipHied (“Realisation of a UnipHied pH scale”) launched an interlaboratory comparison among highly experienced electrochemistry expert laboratories to assign the first such reference ${\mathrm{pH}}_{\mathrm{abs}}^{{\mathrm{H}}_{2}\mathrm{O}}$ values by adopting an extensive statistical treatment of the reported measurement data: to phosphate buffer in water–ethanol mixture (50 wt% of ethanol) and ammonium formate buffer in pure ethanol. Two different measurement setups — one capable of being easily adopted in industrial applications — have been used to demonstrate the robustness of ${\mathrm{pH}}_{\mathrm{abs}}^{{\mathrm{H}}_{2}\mathrm{O}}$ measurement. This is an important step towards wider adoption of the ${\mathrm{pH}}_{\mathrm{abs}}^{{\mathrm{H}}_{2}\mathrm{O}}$ concept in practice, like liquid chromatography, biofuels analysis and electrocatalysis.

**Supplementary Information:**

The online version contains supplementary material available at 10.1007/s00216-023-05043-5.

## Introduction

pH is one of the most widely measured physical–chemical parameters. Its measurement in purely aqueous solutions of limited ionic strength (*I* < 0.1 mol kg^−1^) is straightforward. pH can be traced back to an internationally accepted conventional reference procedure based on potentiometric measurement in a transference-free Harned cell and calculations based on the Debye-Hückel electrolyte solutions theory. [1]

While pH is, in the majority of cases, measured in dilute aqueous solutions, measurements of pH in a broader range of systems (e.g. non-aqueous solutions) are becoming more and more important. One of the solvents attracting attention is ethanol, e.g. in industrial or analytical chemical applications dealing with biofuel acidity [2], lignocellulose deconstruction [3] and other green applications [4].

There are only a few pH standards for organic solvents and their mixtures with water [5, 6], and, more importantly, even if pH measurements can eventually be performed in different solvents or solvent mixtures, each has its own pH scale, so that the pH values are not intercomparable between solvents. A need by the scientific community for an acidity parameter comparable across different media, as well as the corresponding simple and accessible measurement method, is evident in several fields — liquid chromatography, biofuels, and electrocatalysis, to name a few.

This comparability limitation has been overcome through the introduction of a unified pH scale of absolute pH values, ${\mathrm{pH}}_{\mathrm{abs}}^{{\mathrm{H}}_{2}\mathrm{O}}$, and the development of a differential potentiometric measurement method [7, 8], which enables the comparison of pH values measured in different media. Furthermore, this allows pH values measured in diversified media to be expressed in terms of the familiar aqueous pH scale via the assessment of differential values, ${\mathrm{pH}}_{\mathrm{abs}}^{{\mathrm{H}}_{2}\mathrm{O}}$ [9]. Most of the work on ${\mathrm{pH}}_{\mathrm{abs}}^{{\mathrm{H}}_{2}\mathrm{O}}$ has been done using a differential potentiometric method in a symmetric cell [10]. This measurement is rather demanding by requiring skills and using equipment not readily available at most labs.

Thus, besides reference values, a simple and accessible method for ${\mathrm{pH}}_{\mathrm{abs}}^{{\mathrm{H}}_{2}\mathrm{O}}$ measurement is very much needed. An attempt has been made by Matsubara [11, 12], who used an ISFET electrode and the aqueous Ag/AgCl reference electrode and still needed to account for the liquid junction potential. Maximum uncertainty of ${\mathrm{pH}}_{\mathrm{abs}}^{{\mathrm{H}}_{2}\mathrm{O}}$ of 0.37 in pH was reported for water–dimethylformamide mixtures [11, 12].

A convenient potentiometric method using a conventional glass electrode and a double-junction reference electrode with ionic liquid (IL) in the outer compartment has been proposed [13, 14] but not yet sufficiently tested. In addition, there are still no reference ${\mathrm{pH}}_{\mathrm{abs}}^{{\mathrm{H}}_{2}\mathrm{O}}$ values available for non-aqueous or mixed aqueous–organic solutions.

In this work, we present the results of an interlaboratory comparison that was used, on the one hand, to test the robustness of the simple ${\mathrm{pH}}_{\mathrm{abs}}^{{\mathrm{H}}_{2}\mathrm{O}}$ measurement method and, on the other hand, for the assignment of reference ${\mathrm{pH}}_{\mathrm{abs}}^{{\mathrm{H}}_{2}\mathrm{O}}$ values to phosphate buffer in water–ethanol mixture (50 wt% of ethanol) and to ammonium formate buffer in pure ethanol. These samples of different ethanol concentrations were chosen to cover different challenges related to ${\mathrm{pH}}_{\mathrm{abs}}^{{\mathrm{H}}_{2}\mathrm{O}}$ measurements.

## Experimental

### Samples

The equimolal phosphate (15 mmol kg^−1^ Na_2_HPO_4_, 15 mmol kg^−1^ KH_2_PO_4_) buffer solution was prepared by gravimetry by each institute considering as solvent the water–ethanol mixture containing 50 wt% of ethanol. The salts disodium hydrogen phosphate and potassium dihydrogen phosphate (both 15 mmol kg^−1^) were previously dissolved in water before the addition of ethanol (purity > 99.5%). Each lab used its own salts, which were dried for 2 h at 110 °C before use. The water content of the ethanol was determined by density measurement to ensure the 1:1 ratio of the solvents. To make easier the preparation of the buffer solution, all institutes used the same Excel sheet, which contained detailed instructions for the recipe, quality and treatment of the ingredients.

The formate buffer (10 mmol kg^−1^) was also prepared by gravimetry at each institute by dissolving the desired amount of ammonium formate in high-purity ethanol. Each lab used its own salt (purity > 99%) and commercial absolute ethanol (purity > 99.5%).

Each participant (Table S1) was responsible for ensuring the homogeneity of the bottled samples before opening. Additionally, any small remaining inhomogeneities were expected to induce pH changes far below the measurement uncertainty. However, to avoid any possible changes to the analysed solutions, i.e. solvent evaporation, participants were advised to perform the measurements with the buffers directly after preparation or to store the buffers under refrigeration. As unexpected deviations may result from samples being altered with time, the ages of the solutions at the measurement dates were collected and are given in Table S2.

### Measurement procedure

**Fig. 1:**
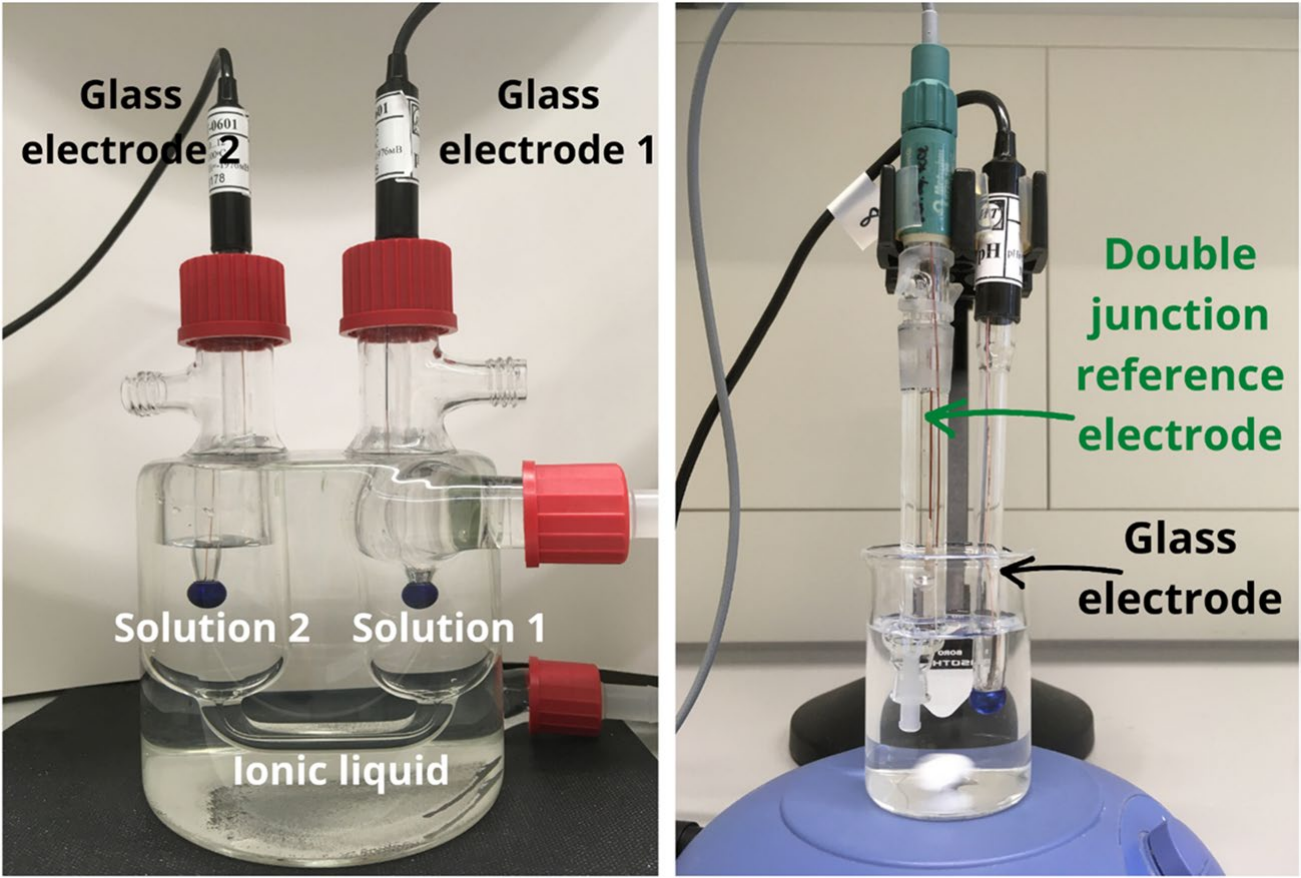
Left: measurement setup for cell I. Right: measurement setup for cell II

Within the European Metrology Research Project “Realisation of a Unified pH Scale” (UnipHied), ${\mathrm{pH}}_{\mathrm{abs}}^{{\mathrm{H}}_{2}\mathrm{O}}$ measurement setups together with measurement procedures have been developed [10]. The measurement procedures are based on the same type of differential potentiometric cell design I (Fig. 1, left) and are collectively referred to as the reference method:

$$\begin{array}{cc}\mathrm{pH\;glass\;electrode\;}2\left|\mathrm{solution\;}2\right|{\mathrm{N}}_{2225}{\mathrm{NTf}}_{2}\left|\mathrm{solution\;}1\right|\mathrm{pH\;glass\;electrode\;}1& \mathrm{I}\end{array}$$

In addition, an easier and more accessible alternative method was used. It consists of reproducing the desired system in 2 pieces: a single glass electrode and an external electrode with a double-junction Ag/AgCl electrode, the inner compartment filled with KCl solution to obtain a stable potential, and the outer compartment filled with ionic liquid triethylpentylammonium bis(trifluoromethylsulfonyl)imide [N_2225_][NTf_2_]. The electrodes are given in Table S3 of the Supplementary material. The same method has been used in an additional study with saline solutions [14] and ethanol buffers [13]. The cell design (Fig. 1, right) is

$$\begin{array}{cc}\mathrm{pH\;glass\;electrode}\left|\mathrm{sample\;or\;standard\;pH\;buffer\;solution}\right| [{\mathrm{N}}_{2225}][{\mathrm{NTf}}_{2}] \left|\mathrm{KClaq}\left|\mathrm{AgCl}\right|\mathrm{Ag}\right.& \mathrm{II}\end{array}$$

The measurement steps with cell II are:

1. Measurement of the difference of potential between the electrodes, Δ*E*, in three standard aqueous buffers (except of lab 4 (two standards) and lab 6 (4 standards)) with known pH values, e.g. buffers with pH 4, 7 and 9. At least two buffers must be used. The number of calibration standards does not affect the quality of the calibration significantly with respect to the measurement uncertainty. Linear regression of pH *vs.* measured difference of potential gives the calibration results expressed as the slope and intercept for the glass electrode.
2. Measurement of the difference of potential between the electrodes, Δ*E'*, in the sample solution, carried out with the same cell II without changing the IL in the outer compartment of the reference electrode.
3. The calculation of ${\mathrm{pH}}_{\mathrm{abs}}^{{\mathrm{H}}_{2}\mathrm{O}}$ values is performed according to Eq. 1:
  1 $${\mathrm{pH}}_{\mathrm{abs}}^{{\mathrm{H}}_{2}\mathrm{O}}=\frac{\left(\Delta E{\prime}-\mathrm{intercept}\right)}{\mathrm{slope}}$$

This procedure follows the multipoint calibration recommended by IUPAC [1]. To validate the measurement method based on cell design II, the samples were also measured with cell I using the independent reference method described in Heering et al. [10]. Using this method, ${\mathrm{pH}}_{\mathrm{abs}}^{{\mathrm{H}}_{2}\mathrm{O}}$ values for phosphate buffer were measured by two institutes, whereas ${\mathrm{pH}}_{\mathrm{abs}}^{{\mathrm{H}}_{2}\mathrm{O}}$ values for formate buffer were obtained by three institutes.

The feasibility of replacing the KCl salt bridge of a double-junction commercial combined pH electrode with ionic liquid was assessed. EtOH-trode electrodes from Metrohm were used. The preliminary tests made after a few weeks of stabilising IL inside the electrode showed that these electrodes are unsuitable for the comparison. The electrodes exhibit a drift (both intercept and slope) with a magnitude 10 times larger than before their modification by introducing IL. Their behaviour is highly affected by an unstable flow from the aqueous inner solution to outer IL. The results do not match the reference ${\mathrm{pH}}_{\mathrm{abs}}^{{\mathrm{H}}_{2}\mathrm{O}}$ values (cell I), and the shift depends on the solvent. The reason is probably the ground-joint diaphragm, which does not allow for sufficient IL leakage. Therefore, combined glass electrodes have been used as glass electrode half-cells, thus measuring only the glass electrode signal against an external double-junction Ag/AgCl reference electrode.

## Results and discussion

**Fig. 2:**
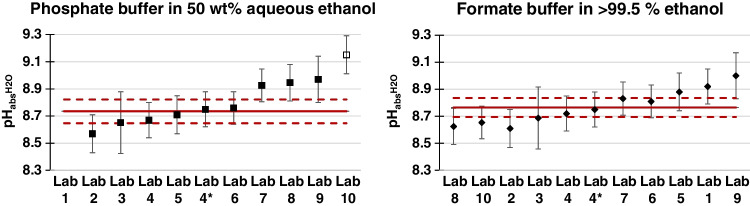
Results with cell II. The proposed interlaboratory comparison reference value (median) is displayed as the solid red line together with its standard measurement uncertainty (dashed red line). Lab 1 used cell I for phosphate buffer, and the obtained value is left out. For phosphate buffer, the result obtained by Lab 10 (open symbol) was not considered in the calculation of the median, as this participant reported problems during solution preparation. Lab4*, electrode from another producer, not used for consensus value

Table S4 summarises the ${\mathrm{pH}}_{\mathrm{abs}}^{{\mathrm{H}}_{2}\mathrm{O}}$ measurement results reported by the participants to interlaboratory comparison exercise for the two analysed matrices. The results are graphically represented in Fig. 2.

### Considerations on uncertainty calculation performed on individual reported ${\mathrm{pH}}_{\mathrm{abs}}^{{\mathrm{H}}_{2}\mathrm{O}}$ values

Except for FC.ID, where a Monte Carlo method was used to calculate measurement uncertainties, all other institutes used the standard GUM approach [15]. It was decided among the participants that all the institutes would consistently take into account a standard uncertainty related to the residual liquid junction potential (RLJP) of 7 mV. This value originates from investigations made by ALU-FR [16] on liquid junctions in aqueous and non-aqueous solutions. It does not consider ionic strength effects nor correlations between calibration and measurement. This uncertainty estimate of (RLJP) to the overall uncertainty is conservative, as the value was obtained as an average from experiments with a number of solvents, most of them more different from water than ethanol. Surely, the effect of RLJP is different for each sample solution, hence demanding further research. As the liquid junction potential dominates the ${\mathrm{pH}}_{\mathrm{abs}}^{{\mathrm{H}}_{2}\mathrm{O}}$ measurement uncertainty, all the reported measurement uncertainties are of comparable magnitude.

### Consensus values of participant results of the interlaboratory comparison and their uncertainties

The phosphate and formate buffers have been prepared individually at each institute, as they can be considered independent. For phosphate buffer, the ${\mathrm{pH}}_{\mathrm{abs}}^{{\mathrm{H}}_{2}\mathrm{O}}$ values reported by Lab 1 and Lab 10 were left out: Lab 1 used cell I instead of cell II and Lab 10 reported problems during solution preparation.

Lab 4 has two results for both buffers, each measured with a different electrode. Only one value, that Lab 4 reported as their interlaboratory comparison result, is used for consensus value. The other value is shown in Fig. 2 as a comparison.

The interlaboratory comparison consensual values involve the consensus of the participating laboratories from the reported results through the median as a more robust estimation than the mean, being less sensitive to outliers.

The consensual ${\mathrm{pH}}_{\mathrm{abs}}^{{\mathrm{H}}_{2}\mathrm{O}}$ values (the median) together with their associated standard uncertainties are 8.74 ± 0.09 (*k* = 1) for phosphate buffer in 50 wt% aqueous ethanol and 8.77 ± 0.07 (*k* = 1) for formate buffer in > 99.5% ethanol. The calculations are described in the Supplementary material.

The standard uncertainties for the ${\mathrm{pH}}_{\mathrm{abs}}^{{\mathrm{H}}_{2}\mathrm{O}}$ assigned to the two samples analysed with cell II are very similar. These uncertainties include all the factors that may vary from one laboratory to another that cannot be evaluated individually. The very close obtained values suggest that the potentiometric alternative method is well implemented by the participants to the interlaboratory comparison and can be attributed to the fact that the applied procedure is clear and complete enough.

Comparison between the consensual ${\mathrm{pH}}_{\mathrm{abs}}^{{\mathrm{H}}_{2}\mathrm{O}}$ values with the reference ${\mathrm{pH}}_{\mathrm{abs}}^{{\mathrm{H}}_{2}\mathrm{O}}$ values from Table 1 allows detection of a possible bias and hence enables to estimate the trueness of the alternative method. A perfect agreement with reference ${\mathrm{pH}}_{\mathrm{abs}}^{{\mathrm{H}}_{2}\mathrm{O}}$ values obtained with the cell I has been found for phosphate buffer despite the fact the preparation of this buffer is more complex as four compounds had to be weighed and the desired solvent ratio exactly be matched. The agreement is poorer for the formate buffer, although suitable as the results remain consistent within the standard uncertainty. The differences in consistency between the samples might be explained by the nature of the solvent. Indeed, pure ethanol buffer solutions seem to be more sensitive to handling. More research is needed to give a full explanation.

**Table 1:** Reference ${\mathrm{pH}}_{\mathrm{abs}}^{{\mathrm{H}}_{2}\mathrm{O}}$ values obtained with cell I and their respective standard uncertainties, *u*

Participant	Phosphate buffer in 50 wt% aqueous ethanol	Formate buffer in > 99.5% ethanol
Lab 4	8.734, *u* = 0.130	8.941, *u* = 0.130
Lab 2	n/a	8.86, *u* = 0.14
Lab 6	8.75, *u* = 0.13	8.87, *u* = 0.13

No evidence could be found for the dependency of ${\mathrm{pH}}_{\mathrm{abs}}^{{\mathrm{H}}_{2}\mathrm{O}}$ measurement results on the age of the sample solutions. Sample ages from 0 to 20 days were investigated. Also, no evidence could be found for the ${\mathrm{pH}}_{\mathrm{abs}}^{{\mathrm{H}}_{2}\mathrm{O}}$ measurement result to depend on the electrode used for the measurements irrespective of the measurement method (cell design I vs. II).

Moreover, results obtained from measurements at 23 °C were found to agree with the other results obtained at 25 °C, indicating the ${\mathrm{pH}}_{\mathrm{abs}}^{{\mathrm{H}}_{2}\mathrm{O}}$ not being significantly temperature-dependent for the matrices investigated in this study. Phosphate buffer with the same composition was analysed (not published results) within the past EURAMET EMRP project “Metrology for Biofuels” using a primary pH system, and the calculated temperature coefficient was about − 0.006 pH/°C. ${\mathrm{pH}}_{\mathrm{abs}}^{{\mathrm{H}}_{2}\mathrm{O}}$ method is, thus, not sensitive enough to highlight such small differences.

## Conclusions

${\mathrm{pH}}_{\mathrm{abs}}^{{\mathrm{H}}_{2}\mathrm{O}}$ Values of two ethanol samples with different ethanol content were measured in an interlaboratory comparison by 10 participants. The consensual values are 8.74 ± 0.09 (*k* = 1) for phosphate buffer in 50% ethanol (median of 8 values) and 8.77 ± 0.07 (*k* = 1) for formate buffer in ethanol (median of 10 values). Results obtained from the measurement method based on cell design II were consistent for both samples.

The interlaboratory comparison involves laboratories that can represent end-users using the ${\mathrm{pH}}_{\mathrm{abs}}^{{\mathrm{H}}_{2}\mathrm{O}}$ measurement according to cell design II, which end-users can easily adopt. The exercise showed that buffered solutions, with assigned ${\mathrm{pH}}_{\mathrm{abs}}^{{\mathrm{H}}_{2}\mathrm{O}}$ values (and associated uncertainty), could be reproducibly prepared for use in routine measurement laboratory. Hence, a simple potentiometric method can be considered suitable for measuring ${\mathrm{pH}}_{\mathrm{abs}}^{{\mathrm{H}}_{2}\mathrm{O}}$ in several industrial applications [2–4]. Additional results obtained from the reference method (cell design I) were found to be consistent with results from the method based on cell design II, independently of the used electrode, thus supporting the robustness of the ${\mathrm{pH}}_{\mathrm{abs}}^{{\mathrm{H}}_{2}\mathrm{O}}$ simple and accessible measurement procedure using cell design II.

### Supplementary Information

Below is the link to the electronic supplementary material.

Supplementary file1 (DOCX 43 KB)
