# Supplementary material for: Rigorous pH measurement in non-aqueous solution: measurement method and reference values in ethanol
Source: Anal Bioanal Chem. 2023 Nov 25;416(2):461–5. doi: 10.1007/s00216-023-05043-5 (PMC10761385; doi:10.1007/s00216-023-05043-5)
Supplement: Supplementary file 1 — Supplementary file1 (DOCX 43 KB) [file 216_2023_5043_MOESM1_ESM.docx]

**Rigorous pH measurement in nonaqueous solution: measurement method and reference values in ethanol**

**Supplementary Information**

Frank Bastkowski, Agnes Heering, Emrah Uysal, Lokman Liv, Ivo Leito, Raquel Quendera, Luís Ribeiro, Lisa Deleebeeck, Alan Snedden, Dániel Nagy, Zsófia Nagyné Szilágyi, Filomena Camões, Bárbara Anes, Matilda Roziková, Daniela Stoica

Contents

[1. Participants 1](#_Toc149051440)

[2. Timetable of measurements 2](#_Toc149051441)

[3. Electrodes 2](#_Toc149051442)

[4. Measurement results as declared by the participants 4](#_Toc149051443)

[5. Evaluating the consensus values of participant results 4](#_Toc149051444)

## Participants

Overall, 10 institutes (7 national metrology institutes and 3 university laboratories) participated in the comparison. Table S 1 lists the participating institutes.

**Table S 1** **List of participants of the interlaboratory comparison**

| **Acronym** | **Institute** | **Country** |
| --- | --- | --- |
| ALU-FR/ Lab 9 | Albert-Ludwigs-Universität Freiburg | Germany |
| BFKH/ Lab 3 | Budapest Főváros Kormányhivatala | Hungary |
| CMI/ Lab 8 | Český Metrologický Institut | Czech Republic |
| DFM/ Lab 4 | Dansk Fundamental Metrologi A/S | Denmark |
| FC.ID/ Lab 5 | FCiências.ID - Associação para a Investigação e Desenvolvimento de Ciências | Portugal |
| IPQ/ Lab 1 | Instituto Português da Qualidade, I.P. | Portugal |
| LNE/ Lab 10 | Laboratoire national de métrologie et d’essais | France |
| PTB/ Lab 2 | Physikalisch-Technische Bundesanstalt | Germany |
| TUBITAK UME/ Lab 7 | The Scientific and Technological Research Council of Turkey-National Metrology Institute | Turkey |
| UT/ Lab 6 | Tartu Ülikool | Estonia |

## Timetable of measurements

Table S 2 lists the sample ages for the three samples for a potential correlation with the measurement result.

**Table S 2** Sample ages at the measurement date in days**^1)^**.

| **Institute** | **Sample age** | |
| --- | --- | --- |
|  | **Phosphate buffer** | **Formate buffer** |
| Lab 1 | 1-2 | 1-2 |
| Lab 2 | 1 | 0 (cell I), 5 (cell II) |
| Lab 3 | 3 | 1 |
| Lab 4 | 1 (cell II), 14 (cell I) | 1 (cell II), 14 (cell I) |
| Lab 5 | 1 | 1 |
| Lab 6 | 0-15 (cell II), 0-17  (cell I) | 0-9 (cell II), 0-7  (cell I) |
| Lab 7 | 1 | 1, 4, 5, 20 |
| Lab 8 | 1-2 | 1-2 |
| Lab 9 | 0 | 0 |
| Lab 10 | 5 | 1 |

^1)^ Sample age at the measurement date (time interval between sample preparation and measurement)

## Electrodes

Cell I measurements were done by Lab 2, Lab 4 and Lab 6 with solid metal-contact half-cell (Izmeritelnaya tekhnika EST-0601).

Electrodes used with cell II are given in Table S 3.

**Table S 3** Electrodes used matching cell design II.

| **Institute** | **Glass electrode** | | **Ag/AgCl reference electrode^1)^** |
| --- | --- | --- | --- |
|  | **Electrode type** | **Brand name** |  |
| Lab3^2)^ | Combined | Metrohm Solvotrode 6.0229.100 | Single junction Metrohm 6.0750.100 |
| Lab 5 | Combined | Metrohm EtOH trode 6.0269.100 | Double junction^1)^ Metrohm 6.0729.100 |
|  |  |  |  |
| Lab 1^3)^ | Single: internal filling | Metrohm 6.0150.100 | Double junction^1)^ Metrohm 6.0729.108 |
| Lab 4^4)^ | Single: internal filling | Radiometer PHG301-9 | Double junction^1)^ Metrohm 6.0729.100 |
| Lab 7 | Single: internal filling | Sentek P10 | Metrohm 6.0729.100 |
|  |  |  |  |
| Lab 2 | Single: metal solid-contact | Izmeritelnaya tekhnika EST-0601 | Double junction^1)^ Metrohm 6.0729.100 |
| Lab 4^4)^ | Single: metal solid-contact | Izmeritelnaya tekhnika EST-0601 | Double junction^1)^ Metrohm 6.0729.100 |
|  |  |  |  |
| Lab 6 | Single: metal solid-contact | Izmeritelnaya tekhnika EST-0601 | Double junction^1)^ Metrohm 6.0729.100 |
| Lab 8 | Single: metal solid-contact | Izmeritelnaya tekhnika EST-0601 | Double junction^1)^ Metrohm 6.0729.100 |
| Lab 10 | Single: metal solid-contact | Izmeritelnaya tekhnika EST-0601 | Double junction^1)^ Metrohm 6.0729.100 |
| Lab 9 | Single: metal solid-contact | Izmeritelnaya tekhnika EST-0601 | Double junction^1)^ Metrohm, 6.0726.110 |

^1)^ For double junction electrodes, the inner chamber is filled with 3 M KCl and the outer chamber is filled with IL N_2225_NTf_2_.

^2)^ Cell design II was realised by filling IL into the capillary, the sample into the sensing electrode part, and 3 M KCl into the reference part of the U-tube cell.

^3)^ In the case of the phosphate buffer, Lab 8 used two metal contact electrodes with IL in the capillary (cell design I), and the results were left out. Calibration of the electrodes was performed using commercial AgAgCl reference electrode Metrohm 6.0733.100.

^4)^ calibration performed using two standards

Results obtained from the glass part of the combined pH electrodes agreed well with results obtained with pH half-cells. The glass part of the combined electrodes has been found to be suitable for buffered and unsuitable for unbuffered ethanol mixtures elsewhere [1, 2]. The leakage of salt bridge electrolyte from the reference electrode compartment of the combined electrode has a larger effect in the case of unbuffered solutions.

One institute realised cell design II by filling IL into the capillary, the sample into the sensing electrode part, and 3 mol dm^-3^ KCl into the reference part of the U-tube cell instead of using a double junction reference electrode. Those measurements also agreed well with other results for both samples.

## Measurement results as declared by the participants

**Table S 4** $pH_{\mathrm{abs}}^{H_{2}O}$ measurement results of the interlaboratory comparison with standard measurement uncertainties as declared by the participants

| **Participant** | $pH_{\mathrm{abs}}^{H_{2}O}$ values reported for **phosphate buffer** | $pH_{\mathrm{abs}}^{H_{2}O}$ values reported for **formate buffer** |
| --- | --- | --- |
| Lab 1 | n/a | 8.92 ± 0.13 |
| Lab 2 | 8.57 ± 0.14 | 8.61 ± 0.14 |
| Lab 3 | 8.652 ± 0.227 | 8.687 ± 0.229 |
| Lab 4 | 8.67 ± 0.13 | 8.72 ± 0.13 |
| Lab 4*^a^* | 8.75 ± 0.13 | 8.75 ± 0.13 |
| Lab 5 | 8.71 ± 0.14 | 8.88 ± 0.14 |
| Lab 6 | 8.76 ± 0.12 | 8.81 ± 0.12 |
| Lab 7 | 8.926 ± 0.121 | 8.831 ± 0.123 |
| Lab 8 | 8.946 ± 0.134 | 8.625 ± 0.134 |
| Lab 9 | 8.97 ± 0.17 | 9.00 ± 0.17 |
| Lab 10*^b^* | 9.151 ± 0.140 | 8.654 ± 0.120 |

*^a^* Different glass electrode was used, not used for consensus value. *^b^* For phosphate buffer, the result obtained by Lab 10 was not considered in the calculation of the reference value (consensus value of participant results) as this participant reported problems during solution preparation.

## Evaluating the consensus values of participant results and their uncertainties

For a population with an odd number of results, i.e. formate buffer, the median is the middle number, whereas for those with an even number of data, i.e. phosphate buffer, it represents the mean of the two middle values. Under the hypothesis of a normal distribution, also called Gaussian distribution, of the results, the standard deviation of the median, *u*(m), is calculated with equation 1:

| $u\left( m \right)= \frac{1.858}{\sqrt{N-1}}\cdot MAD$ | (1) |
| --- | --- |

With MAD being the median of the absolute deviations and defined by equation 2:

| $MAD=med\left\{ \left\vert x_{i}-m \right\vert\right\}$ | (2) |
| --- | --- |

with *i* = 1, 2 …, *N*, where *N* represents the number of reported results. *m* accounts for the calculated median value.

**Table S 5** Interlaboratory comparison reference $\mathrm{pH}_{\mathrm{abs}}^{H_{2}O}$values (consensus values of participant results), their respective standard uncertainties *u*, numbers *N* of included participant results and the respective observed and critical *χ*^2^ values

|  | **Phosphate buffer in 50 wt% aqueous ethanol** | **Formate buffer in >99.5 % ethanol** |
| --- | --- | --- |
| **Estimator** | 8.74, *u* = 0.09 | 8.77, *u* = 0.07 |
| *N* | 8*^a^* | 10 |
| $\chi_{obs}^{2}$ | 8 | 8 |
| $\chi_{crit}^{2}$ | 17 | 18 |

*^a^* Values reported by Lab 1 and Lab 10 were left out

Further, a consistency test has been made to verify whether the results reported by the participants are consistent with the estimated uncertainty of the reference value (obtained as the consensus value of participant results). For this purpose, the chi-square parameter,
$\chi_{obs}^{2}$, was calculated with equation 3:

| $\chi_{obs}^{2}=\sum\left[ \frac{{(x}_{i}-m)}{u(x_{i})} \right]^{2}$ | (3) |
| --- | --- |

$\chi_{obs}^{2}$ was compared with the critical value,
$\chi_{crit}^{2}$_,_ for a significance level of 0.05 and *N*-1 degrees of freedom. Table S 5 shows that all data used for the statistical treatment passed the chi-squared test since the calculated values do not exceed the critical ones. This finding validates the consistency hypothesis and suggests that the uncertainties of the consensus estimates describe well the observed dispersion of the reported $\mathrm{pH}_{\mathrm{abs}}^{H_{2}O}$ results for the two analysed samples.

**References**

[1] Heering A., Bastkowski F., Seitz S., Glass electrode half-cells for measuring unified pH in ethanol-water mixtures, J. Sens. Sens. Syst. 2020;9:383–89.

[2] Deleebeeck L., Snedden A., Nagy D., Roziková M., Heering A., Bastkowski F., Leito I., Quendera R., Cabral V., Stoica D., Unified pH measurements of ethanol, methanol, and acetonitrile, and their mixtures with water, Sensors. 2021;21:3935.
